# Identification and validation of a prognostic model of necroptosis-related lncRNAs in hepatocellular carcinoma

**DOI:** 10.3389/fgene.2022.907859

**Published:** 2022-09-29

**Authors:** Min Chen, Guang-Bo Wu, Shan Hua, Zhi-Feng Zhao, Hong-Jie Li, Meng Luo

**Affiliations:** ^1^ Department of General Surgery, Shanghai Ninth People’s Hospital Affiliated to Shanghai Jiao Tong University School of Medicine, Shanghai, China; ^2^ Department of Urology, Shanghai General Hospital, Shanghai Jiao Tong University School of Medicine, Shanghai, China

**Keywords:** hepatocellular carcinoma, prognostic model, necroptosis, lncRNA, NRAV

## Abstract

**Background:** The study focused on establishing a prognostic survival model with six necroptosis-related lncRNAs to predict overall survival (OS) in patients with hepatocellular carcinoma (HCC).

**Methods:** The data of gene expression and clinical information of HCC patients were obtained from The Cancer Genome Atlas (TCGA). Cox regression with LASSO was used for constructing a necroptosis-related lncRNA survival model, which we further validated with qRT-PCR *in vitro*. The relative bioinformatics analysis and consensus cluster analysis were performed based on six differentially expressed lncRNAs.

**Results:** The survival prognostic model was constructed by using data from TCGA. Receiver operating characteristic (ROC) curves showed a good survival prediction by this model. GSEA showed that several signaling pathways were related to HCC progression. Immune-related functional analysis showed that aDCs, macrophages, Th2 cells, and Tregs have stronger correlation with the high-risk group. The consensus cluster analysis further validated the 6-lncRNA prognostic model.

**Conclusion:** A novel 6-lncRNA (AL606489.1, NRAV, LINC02870, DUXAP8, “ZFPM2-AS1,” and AL031985.3) prognostic model had an accurately predictive power in HCC prognosis, which might be worthy of clinical application.

## Introduction

According to the latest estimates made by the International Agency for Research on Cancer [IARC (http://gco.iarc.fr)], hepatocellular carcinoma (HCC), one of the most general subtypes of primary hepatocellular carcinoma, is the major cause of cancer-associated death and is gradually becoming a public health problem worldwide (Llovet et al., 2021). In recent years, it has been demonstrated that the incidence of HCC in the United States has increased from that in previous studies (Ashhab et al., 2019; Kim and El-Serag, 2019). Meanwhile, a rapid increase in the incidence of HCC was also observed, attributable to significant hepatitis B virus infection rates (Wei et al., 2020).

Despite promising advances such as partial hepatectomy, liver transplantation, interventional surgery, and systemic treatment in treatment of HCC, the long-term outcome of late-stage HCC remains ineffective and essentially palliative (Hartke et al., 2017; Zhao et al., 2020). Until now, relatively vague predictions have been provided from extensively applied staging systems in evaluating the options for treatment and prognostic outcomes for patients with HCC (Cao et al., 2014; Yoo et al., 2018). However, at present, there is still a lack of sensitive and highly specific biomarkers and tumor signature able to predict prognosis of HCC patients. Therefore, it is imperative to identify novel prognostic biomarkers and signature to accurately predict both the improvement and overall survival (OS) of HCC patients (Steyerberg et al., 2013).

Necroptosis, also named as programmed necrosis, is identified as a new form of cell death that is characterized by increasing inflammation through the release of damage‐associated molecular patterns (DAMPs) from necroptotic cells (Newton and Manning, 2016). Necroptosis could be activated by activation of receptor-interacting serine–threonine protein kinase 1 (RIP1), RIP3, and mixed lineage kinase domain-like (MLKL) to execute cell death as an alternative mode of cell death (Khan et al., 2021). Previous studies have shown that necroptosis is associated with the occurrence and development of various cancers and tumor-related signaling pathways in pancreatic carcinoma, prostate carcinoma, head and neck squamous cell carcinoma, liver disease, etc. (Xie et al., 2017; Li et al., 2020; Xue et al., 2020; Lee et al., 2021). In addition, a direct role of necroptosis in HCC has also been studied. The repression of necroptosis in all common human hepatoma cell lines like Huh-7, HepG2, and Hep3B was observed, which suggested this cell death mechanism might play an important role in malignant transformation of tumor cells (Koo et al., 2015).

Long non-coding RNA (lncRNA) is a class of non-encoding RNA molecules with a transcript length of more than 200 nucleotides. The mechanism of lncRNA includes gene regulation in *cis* or in *trans* and regulation of their interacting proteins, which are involved in regulating various aspects of tumors (Kung and Lee, 2013; Tran et al., 2016; Kopp and Mendell, 2018). It is well-known that lncRNAs regulate various aspects of tumors, especially in the progression and prognosis of liver cancer (Bhan et al., 2017; Pan et al., 2019). However, few studies have focused on the relationship or mechanism between the expression of lncRNA and necroptosis with development of HCC.

Therefore, in this study, the mRNA transcriptomic data and clinical information of HCC patients were obtained from TCGA for constructing and validating the HCC prognostic signature based on the necroptosis-related lncRNA. Meanwhile, a series of bioinformatics analyses, including infiltrating immune cells, gene set enrichment analysis, immune checkpoints, and chemotherapy drug analysis, were performed to understand the necroptosis-related lncRNA expression and its potential impact in development of HCC. The results of this scenario helped determine potential biomarkers that might be novel therapeutic targets for HCC.

## Materials and methods

### Datasets

The clinical information and FPKM-masked sequencing data (374 HCC tissues and 50 normal tissues) were retrieved from The Cancer Genome Atlas (TCGA; https://portal.gdc.cancer.gov). The sequencing data were further annotated with human gene annotation files from the Ensembl official website (http://asia.ensembl.org/index.html). Around 67 necroptosis-related genes were systematically searched and chosen according to the references from the representative necroptosis-related literature and presented in Supplementary Material S1. The main codes of this research are presented in Supplementary Material S4.

### Identification of necroptosis-related lncRNA

First, we selected the necroptosis-related lncRNAs (NRlncs) by calculating the correlation coefficient between the expression levels of necroptosis-related genes (NRG) and the expression level of lncRNA. We defined the filter criteria as |Pearson r| > 0.4 and *p* < 0.001 and selected the one that met the criteria as the NRlncs. Then, the expression of NRlncs was compared between the HCC tissue and normal tissue and further selected 769 NRlncs with standard of LogFC = 1 and false discovery rate (FDR) < 0.05.

### Construction and validation of the prognostic model

To assess the predictive utility of the NRlncs, we randomly divided the HCC tissue equally into training (*n* = 187) and testing groups (*n* = 187) once. Univariate Cox regression analysis was applied and identified 165 prognostic NRlncs with *p* < 0.05 between the HCC and normal groups with the help of the limma R package. The least absolute shrinkage and selection operator (Lasso), a penalized shrunken regression method to avoid overfitting, was applied to select the NRlncs for model construction and, penalty parameter (*λ*) adjustment was performed by cross-validation based on minimum criteria. In this study, six prognostic NRlncs participated in model construction were chosen by using LASSO Cox regression analysis and optimized by cross-validation. The network between prognostic NRlncs and NRG is visualized by R packages “ggplot2” and “ggalluvial.” Every patient’s risk score was calculated based on the formula below: ∑N i = 1(Coef_i_*X_i_). Coef_i_ represented the coefficient of the corresponding NRlncs and the Cox regression model, and X_i_ is the expression level of the NRlncs. Based on the median risk score, patients were divided into two groups: low-risk (<median) and high-risk (≥median). T-distributed stochastic neighbor embedding (t-SNE), principal component analysis (PCA), and analysis of corresponding survival visualization were performed by R packages “pheatmap” and “ggbiplot”.

Then, Kaplan–Meier (K-M) survival curve analysis was introduced to compare the survival rates between high- and low-risk groups by using the R packages “survival” and “survminer.” The time-dependent receiver operating characteristic (ROC) curve analysis was performed using the R package “timeROC” to evaluate the predictive accuracy of this signature and the areas under the curve (AUC) at 1, 3, and 5 years were calculated. Meanwhile, the K-M plot in patients with different clinical characteristics was conducted to check the consistent predictions of the 6-NRlnc signature.

### Construction of nomogram and gene set enrichment analysis

The nomogram prediction model for predicting the 1-, 3-, and 5-year OS of HCC was constructed based on the 6-NRlnc signature risk score and independent clinical factors by using the “rms” R package to improve the clinical application of the prognostic signature. The calibration curves were constructed to assess the nomogram’s accuracy. By using GSEA 4.0.1 software, the Gene set enrichment analyses (GSEA) were performed to investigate the KEGG pathways between the high- and low risk groups with filter criteria of *p* < 0.05 and FDR <5%.

### Immune infiltration and assessing

In order to explore the association between immune cell infiltration and the expression level of 6 NRlncs, various methods or software, including XCELL, TIMER, QUANTISEQ, MCPCOUNTER, EPIC, CIBERSORT-ABS, and CIBERSORT, were involved to count the infiltrating immune cells levels in HCC patients from TCGA. Then, infiltrating immune cells and immune-related function were quantified by single-sample gene set enrichment analysis (ssGSEA) to explore the immune association between the high- and low-risk groups with the help of R packages (“reshape2” and “ggpubr”). Meanwhile, immune checkpoint analysis was conducted to explore the differences in immune checkpoint expression levels between the high- and low-risk groups by R packages (“ggplot2” and “ggpubr").

### Evaluation of chemotherapeutic agent sensitivity

The R package of pRRophetic downloaded from “https://github.com/paulgeeleher/pRRophetic” was involved to predict the half-maximal inhibitory concentration (IC_50_) of chemotherapeutic agents for each patient between the high- and low-risk groups. As for too much chemotherapeutic drug was screened based on six NRlncs, we chose five commonly used drugs in clinical settings for further research.

### Consensus clustering of prognostic NRlncs

The consensus clustering analysis was performed using the R package (“ConsensusClusterPlus”) based on six NRlncs. We, then, determined the optimal tradeoff between stability and informativeness of each cluster based on the similarity between the expression levels of prognostic NRlncs and the proportion of fuzzy similarity measurements. The analysis of K-M survival curve, Sankey diagram, PCA, t-SNE, immune infiltration, immune checkpoints, and chemotherapeutic agent sensitivity was conducted in the similar way of method mentioned above.

### Cell culture

The Hep3B, HepG2 cell line (Human HCC cell), and HL7702 (Human Normal Cells) were purchased from the Chinese Academy of Medical Sciences (Beijing, China). The Hep3B cells were grown in high-glucose Dulbecco’s modified Eagle’s medium (DMEM) supplemented with 10% fetal bovine serum (Life Technologies, Inc., Carlsbad, CA, United States), 1 mM sodium pyruvate, 0.1 mM non-essential amino acids, and 2 mM L-glutamine at 37°C and 5% CO_2_ in a humidified incubator. The HepG2 and HL7702 cells were cultured in RPMI-1640 medium (Gibco, Rockville, MD, United States) supplemented with 10% fetal bovine serum (Life Technologies, Inc., Carlsbad, CA, United States) at 37°C in a humidified 5% CO_2_ atmosphere.

### Quantitative real-time PCR

Here, we used Hep3B, HepG2, and HL7702 to verify the expression of prognostic genes. The total RNA was isolated by TRIzol reagent (Life Technologies Corporation, Carlsbad, CA, United States) under the construction producer’s directions. Then, 0.8 μg mRNA was used for synthesis of 20 μl cDNA using Superscript II reverse transcriptase and random hexamers (Invitrogen, Carlsbad, CA, United States). qRT-PCR was further performed on an ABI Prism 7300 Sequence Detection System with SYBR Green PCR Master Mix (Applied Biosystems). The primers used in this study were AL606489.1 (forward 5′- CTA​AGA​ACT​CAG​GAA​AGA​CAT​CAG​C-3′, reverse 5′- CAT​GGG​ACT​GAA​AGG​GCA​AAG-3′), NRAV (forward 5′- CCG​AGC​AAC​ACC​TAA​ACA​AAA​G-3′, reverse 5′- CGACGATGCTTAGTGICTCCAG-3′), LINCO2870 (forward 5′- GGC​ACC​CAG​GAT​AAC​TCA​GTA​CAC-3′, reverse 5′- GAT​GAG​AAA​TGG​GCG​ACG​TG-3′), DUXAP8 (forward 5′- CITTATCTGTGGATGAACAGGCTAG-3′, reverse 5′- GGC​AGT​CTC​AGA​AGG​AAT​CAG​TG-3′), ZFPM2-AS1 (forward 5′- TGC​AAA​GAT​GAA​CTA​TGA​AGA​GCA​C-3′, reverse 5′- CAAGTICCTCACTGGCTTATTCAG-3′), and AL031985.3 (forward 5′- GCC​AAA​ATT​GTC​CCT​GGT​TG-3′, reverse 5′- TGA​GCC​AAA​CGA​AAC​CTA​ACA​G-3′). GAPDH was the internal comparison gene. The lncRNA expression of relative genes was calculated using the 2−ΔΔCt method with normalization to GAPDH expression.

Meanwhile, siRNA DUXAP8 and siRNA negative control (Thermo Scientific, CA, United States) were transfected with Lipofectamine 2000 reagent (Invitrogen, CA, United States). All the siRNA primer sequences are listed in Supplementary Material S3.

### Western blotting

The whole cell lysates (Beyotime, Shanghai, China) were used to harvest total protein whose protein concentrations were measured by the BCA™ Protein assay kit (Pierce, Appleton, WI, United States). Proteins were separated by SDS-PAGE using 10% acrylamide gels. After three washes in TBST, the membranes were incubated with appropriate dilutions of primary antibodies against *Ki-67* (1:1000; Abcam, Cambridge, MA, United States), *PCNA* (1:1500; Abcam, Cambridge, MA, United States), and *GAPDH* (1:1500; Abcam, Cambridge, MA, United States).

### Cell counting Kit-8

CCK-8 assay was used to measure the viability of Hep3B under various si-DUXAP8 concentrations. The cells were resuspended, seeded in a 96-well plate (6 × 104 cells/well), and cultured in an appropriate environment (37°C, 5% CO_2_.) for 24, 48, 72, and 96 h. Then, they were incubated for 2 h with 10 µl CCK-8 solution (Yeasen, Shanghai, China). The absorbance of each well was measured at 450 nm by a microplate reader (Bio-Rad, Hercules, CA).

### Tissue samples

About 15 HCC samples and adjacent normal tissues were obtained from patients who received surgery in Shanghai Ninth People’s Hospital from 2021 to 2022. All patients signed informed consents, and the specimens were stored in liquid nitrogen at −196 °C for RNA extraction. The collection and use of patient tissue samples were approved by the Ethics Committee of Shanghai Ninth People’s Hospital.

## Result

### Screening for the prognostic necroptosis-related lncRNA genes

**FIGURE 1:**
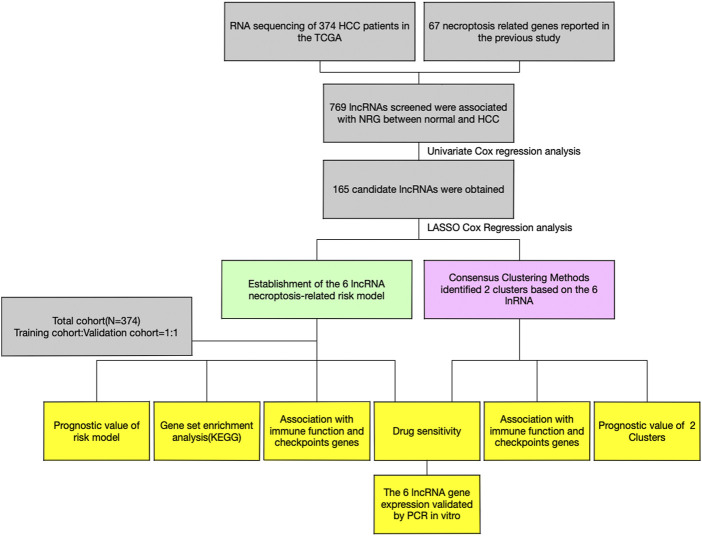
Study flow chart of this research.

**FIGURE 2:**
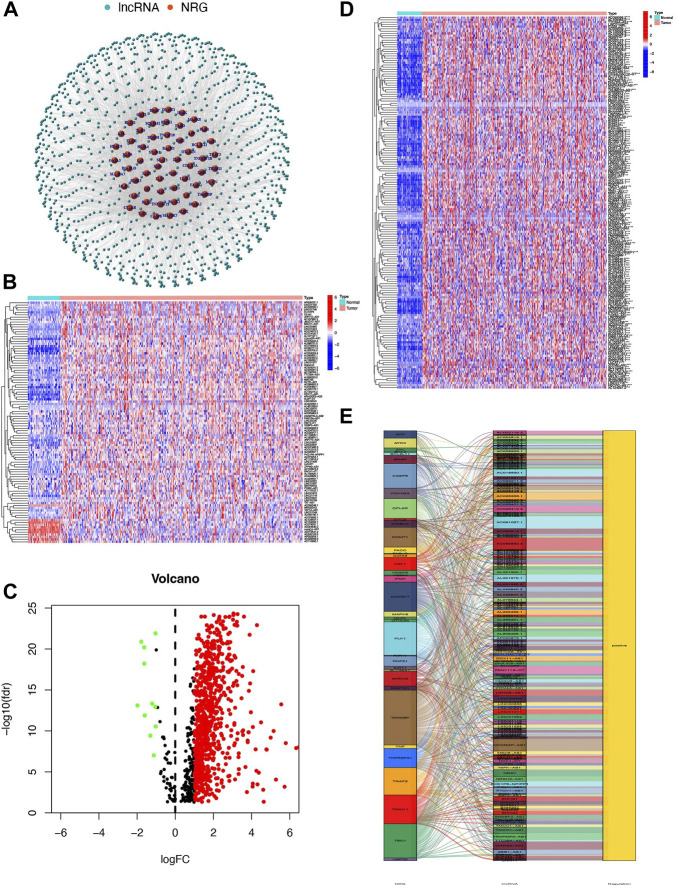
Identification of NRlncs and prognostic NRlncs. **(A)** 769 NRlncs and 60 NRGs were screened out between the normal tissue and HCC. **(B)** Heatmap of NRLncs between the normal tissue and HCC (LogFC = 1, FDR<0.05, *n* = 100). **(C)** Volcano map of NRLncs between the normal tissue and HCC. **(D)** Heatmap of 165 prognostic NRlncs between the normal tissue and HCC. **(E)** Network between prognostic NRlncs and NRG by the ggalluvial R package. *** represented *p* < 0.001.

The research was performed in the procedure depicted in Figure 1. A total of 1016 lncRNAs were screened out and significantly correlated with 60 necroptosis-related genes in the TCGA dataset and were defined as NRlncs and NRG (Figure 2A). About 11 NRlncs were significantly downregulated and 758 were upregulated between normal tissue and HCC (Figures 2B and C, Supplementary Material S2). Next, we conducted a univariate Cox regression analysis and found 165 NRlncs had a strong connection with overall survival (OS) of HCC. The 165 NRlncs of HCC are represented at a higher expression level than the normal tissue (Figure 2D). In order to depict the connections between the 165 NRlncs and related NRG, we determined and visualized the NRlnc co-expressed with the NRG using a co-expression network (Figure 2E).

### Establishment of a prognostic signature based on six prognostic NRlncs

**FIGURE 3:**
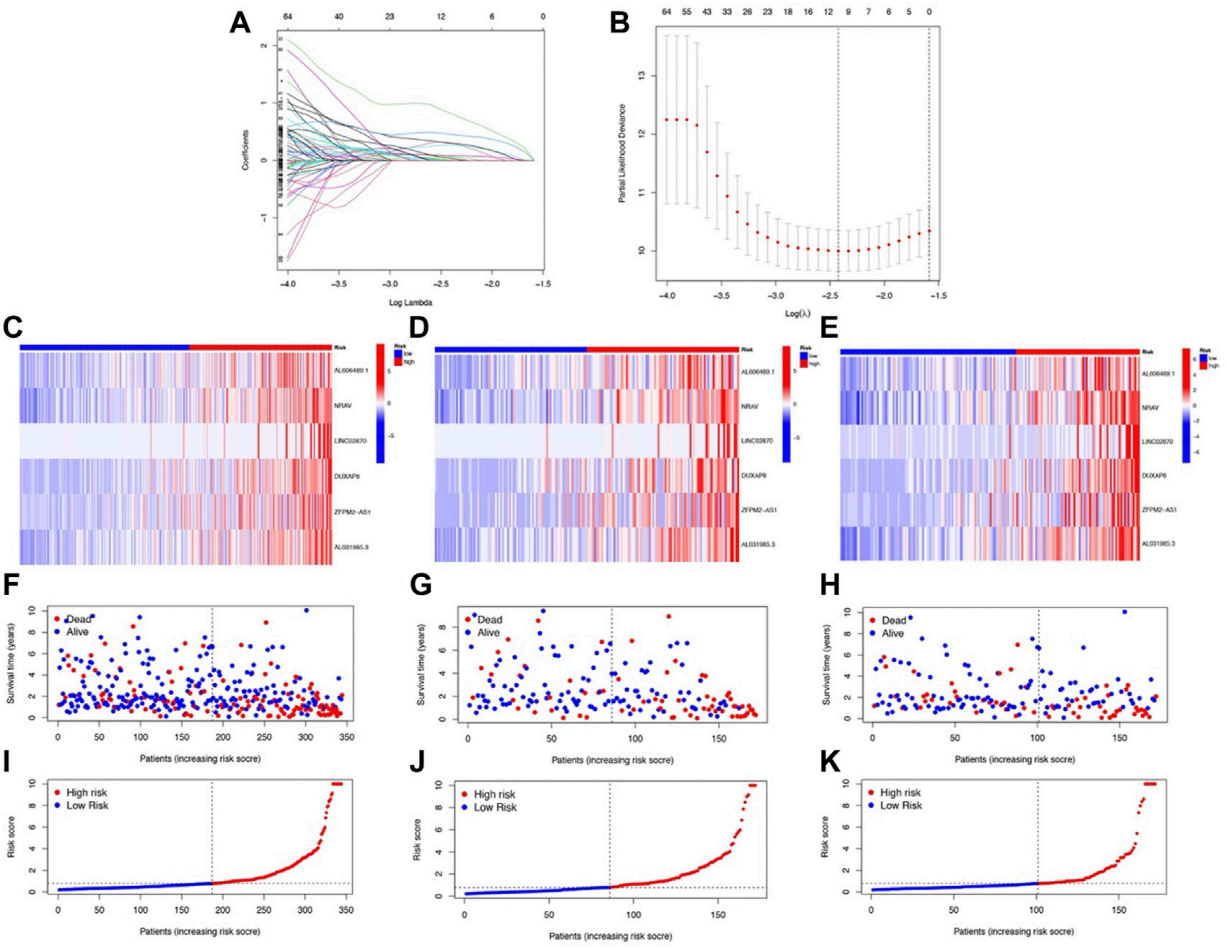
Development and validation of NRlnc signature. **(A)** Partial likelihood deviance for different numbers of variables. **(B)** LASSO Cox regression analysis of 165 NRlncs. The heatmap of the six prognostic NRlncs in **(C)** the TCGA dataset, **(D)** training cohort, and **(E)** testing cohort. The distribution of OS status in **(F)** the TCGA dataset, **(G)** training cohort, and **(H)** testing cohort. The median value and distribution of the risk scores in **(I)** the TCGA dataset, **(J)** training cohort, and **(K)** testing cohort.

Six prognostic NRlncs (AL606489.1, NRAV, LINC02870, DUXAP8, “ZFPM2-AS1,” and AL031985.3) were selected out and used for establishing a prognostic signature using LASSO Cox regression analysis (Figures 3A and B). The six-NRlnc expression could divide the high- and low-risk groups. The formula, risk score = 0.42374403401908 * AL606489.1 + 0.48111958676462 * NRAV +0.438821562947673 * LINC02870 + 1.3664982554004 * DUXAP8 + 0.261088654957603 * ‘ZFPM2-AS1’ + 0.723038773112586 * AL031985.3, was used to compute the risk score of each patient. The six-NRlnc expression could relatively divide the entire patients into high- and low-risk groups in the TCGA dataset, training cohort, and testing cohort (Figures 3C–E). We could also divide patients in the TCGA, training cohort, and testing cohort into two groups by using the median risk scores (Figures 3I–K). In addition, the scatter chart indicated that patients in high-risk groups may have a worse outcome, which was evidenced by the red dots being denser in the high-risk area in the TCGA, training cohort, and testing cohort (Figures 3F–H).

### Prognostic implication of the 6-NRlnc prognostic model

**FIGURE 4:**
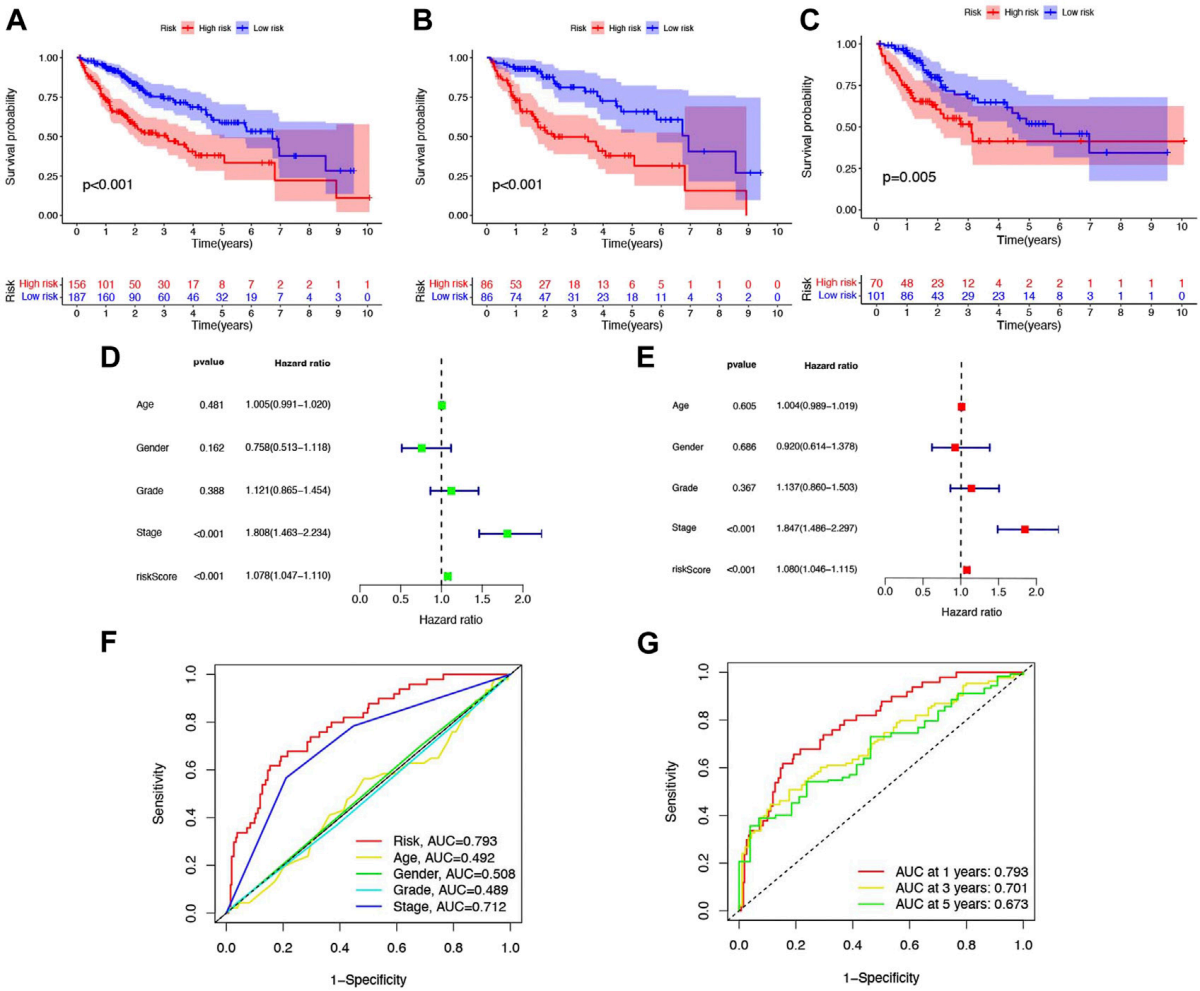
Six-NRlnc signature predicts the efficiency of OS in patients with HCC. Kaplan–Meier curves for OS of patients in the high- and low-risk groups in **(A)** TCGA, **(B)** training cohort, and **(C)** testing cohort. **(D)** OS-related factors screened by univariate Cox regression were analyzed in TCGA. **(E)** OS-related factors screened by multivariate Cox regression were analyzed in TCGA. **(F)** AUC time-dependent ROC curves for different clinical features in TCGA. **(G)** AUC time-dependent ROC curves for OS at 1, 3, and 5 years in TCGA.

From Figures 4A–C, we could know that patients with higher risk scores were more likely to suffer from a markedly shorter OS than their counterpart, which is verified in TCGA, training cohort, and testing cohort. Meanwhile, the risk score would be a significant variable factor for predicting prognosis by the univariate Cox regression analyses (HR = 1.080; CI = 1.046–1.115; *p* < 0.001) and the multivariate survival analyses (HR = 1.078; CI = 1.047–1.110; *p* < 0.001) (Figures 4D,E). The area under ROC (AUC) of the different clinical features also demonstrated that the risk score could have the highest predictable value (AUC = 0.793) among other traditional features, including stage, age, gender, and grade (Figure 4F). Time-dependent ROC analysis was used to investigate the predictive ability of the risk score for HCC patients and found that AUC was 0.793, 0.701, and 0.673 at 1, 3, and 5 years respectively, in TCGA (Figure 4G).

### Clinical implications of the 6-NRlnc prognostic model

**FIGURE 5:**
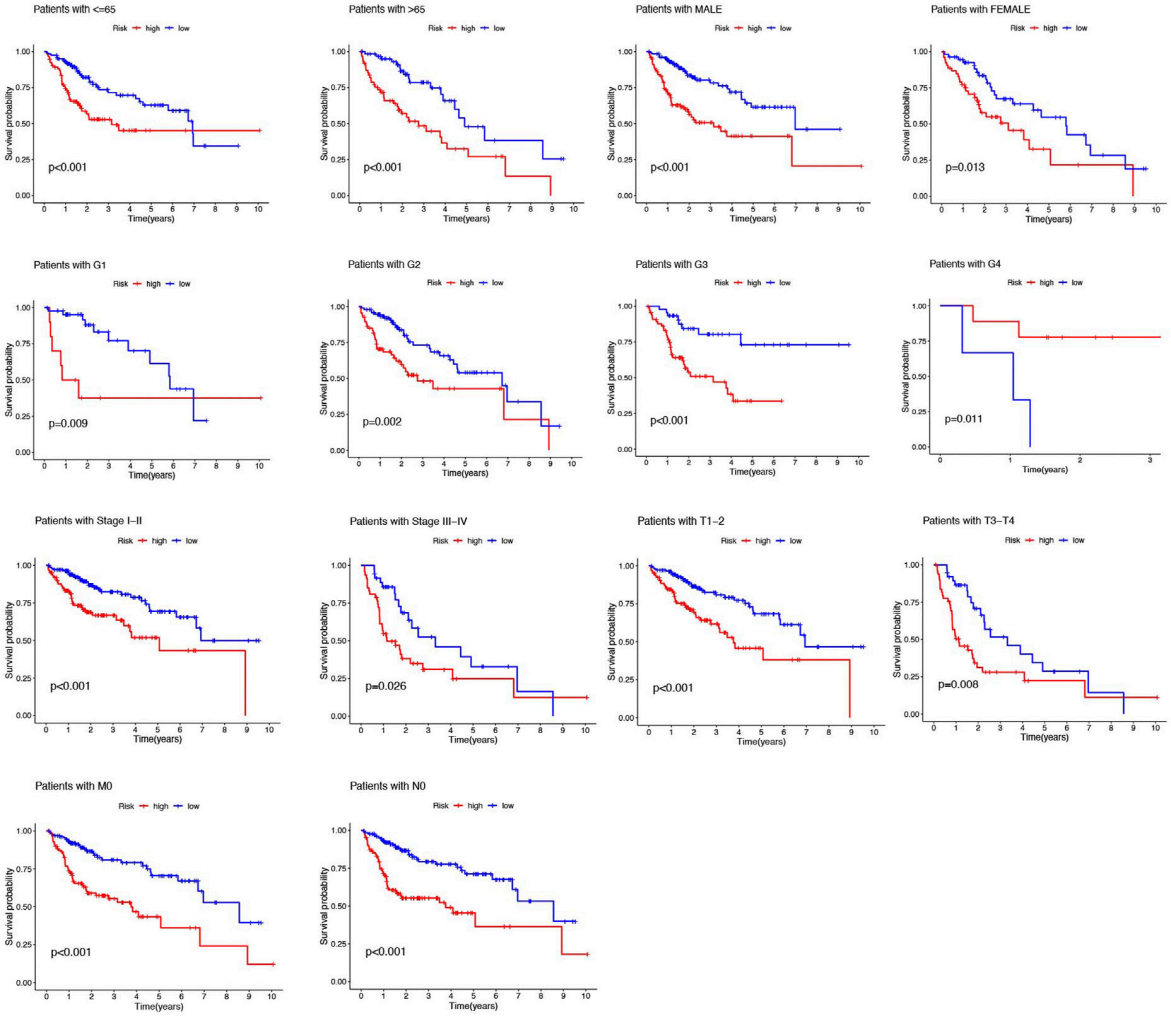
Kaplan–Meier analyses for high- and low-risk groups by age, gender, grade, and stage.

In addition, the HCC patients were divided into distinct subgroups according to age, sex, stage, and clinical grade to further explore the signature’s prognostic value arranged by clinical variables. Figure 5 demonstrated that the survival probabilities of various clinical variables in the high-risk group were worse than those in the low-risk group in nearly all groups, except G4, because of the inadequate number of patients (Figure 5).

**FIGURE 6:**
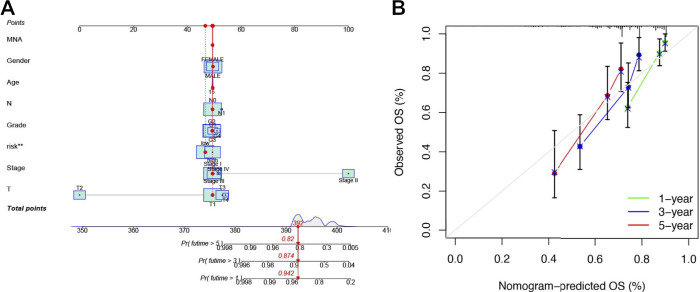
Establishing a nomogram of six-NRlnc signature for predicting prognosis of HCC patients **(A)** Nomogram for predicting HCC patients’ survival. **(B)** Calibration plots applied for predicting the 1-, 3-, and 5-year OS in TCGA.

Then, we further constructed a nomogram to predict the 1-year, 3-year, and 5-year OS of HCC patients according to the fundamental prognostic factors from the multivariate Cox regression analysis. Based on the scores of all the variables in the nomogram, each patient could get a point after being calculated. By drawing a vertical line from the total point to the survival prediction axis, each patient could be predicted the 1-, 3-, and 5-year OS (Figure 6A). From Figure 6B, we could find the nomogram had precise predictive ability when predicting the OS of HCC patients at 1 year, 3 year, and 5 years.

### Signaling pathways in high- and low-risk groups

**FIGURE 7:**
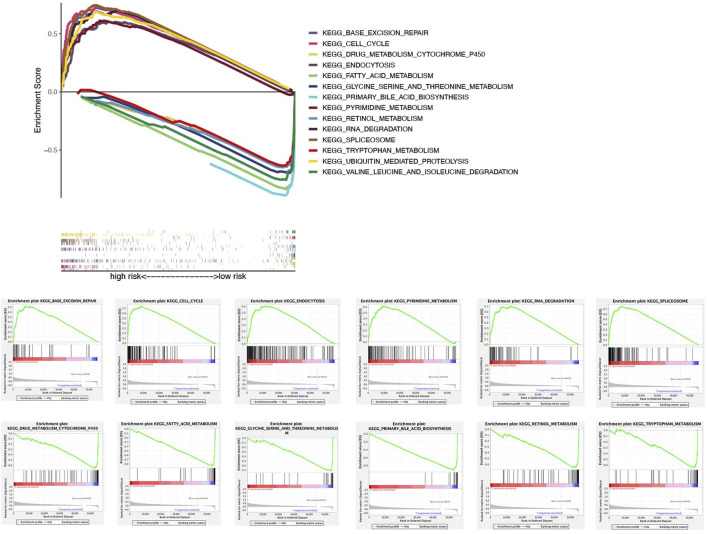
Gene set enrichment analysis of biological functions and pathways.

In the TCGA dataset, ssGSEA showed that signaling pathways, such as BASE_EXCISION_REPAIR (normalized enrichment score NES = 1.96, *p* < 0.05), CELL_CYCLE (NES = 2.06, *p* < 0.05), 、ENDOCYTOSIS (NES = 1.95, *p* < 0.05), PYRIMIDINE_METABOLISM (NES = 1.98, *p* < 0.05), RNA_DEGRADATION (NES = 1.94, *p* < 0.05), SPLICEOSOME (NES = 1.95, *p* < 0.05), and UBIQUITIN_MEDIATED_PROTEOLYSIS(NES = 2.03, *p* < 0.05) in the high-risk group were markedly enriched, while in the low-risk group, signaling pathways, such as DRUG_METABOLISM_CYTOCHROME_P450 (NES = −1.94, *p* < 0.05), FATTY_ACID_METABOLISM (NES = −2.07, *p* < 0.05), GLYCINE_SERINE_AND_THREONINE_METABOLISM (NES = −1.86, *p* < 0.05), PRIMARY_BILE_ACID_BIOSYNTHESIS (NES = −2.01, *p* < 0.05), RETINOL_METABOLISM (NES = −1.95, *p* < 0.05), TRYPTOPHAN_METABOLISM (NES = −1.81, *p* < 0.05), and VALINE_LEUCINE_AND_ISOLEUCINE_DEGRADATION (NES = −1.87, *p* < 0.05) were significantly enriched (Figure 7).

### Tumor immune cell infiltration and gene expression

**FIGURE 8:**
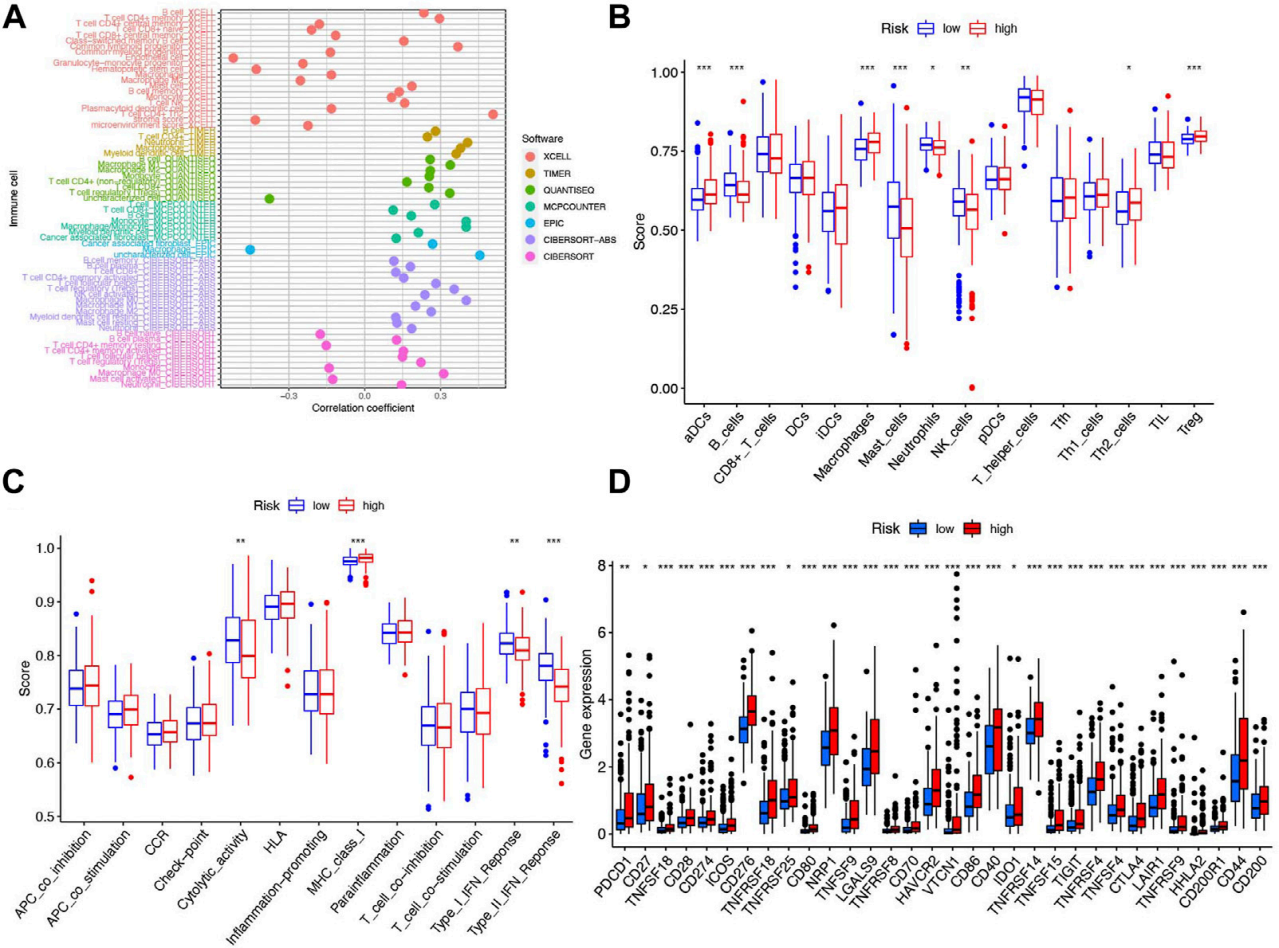
Immune cells and functions based on patient risk scores between high- and low-risk groups in the TCGA cohort. **(A)** Correlation coefficient of immune cells in seven softwares. **(B)** Boxplots showed the cores of 16 immune cells. **(C)** Boxplots showed the cores of 13 immune-related functions. **(D)** Box plot of 32 immune checkpoints in the entire cohort. ***p < 0.001, **p < 0.01, and *p < 0.05.

Here, we investigated the relationship between various immune cells and risk scores of patients in TCGA. From the results of the seven-software (XCELL, TIMER, QUANTISEQ, MCPCOUNTER, EPIC, CIBERSORT-ABS, and CIBERSORT) analysis, we observed most kinds of B-cell, T-cell, macrophage, mast cell, etc. were consistent with the increase of patients’ risk (Figure 8A). To further clarify the difference between the high- and low-risk groups, we conducted ssGSEA analysis to compare the ssGSEA scores between the different risk groups. Here, we found that activated dendritic cells (aDCs), macrophages, T helper 2 cells (Th2 cells) and regulatory T-cells (Tregs) have stronger correlation with the high-risk group while B-cells, mast cells, neutrophils, and natural killer cells (NK cells) were indicated to connect with the low-risk group (Figure 8B, *p* < 0.05). Furthermore, the immune functions between the two groups were also compared to explore the potential immune pathway participating in differentiating the two groups. We found cytolytic activity and MHC (major histocompatibility complex) class I were more active in the high-risk group, while Type I and Type II IFN responses were more active in the low-risk group (Figure 8C, *p* < 0.05). Considering the significance of immune checkpoints in immunotherapies, we compared immune checkpoint expression between the two risk groups. Figure 8D shows that most of these genes, for example, PDCD-1, CD27, TNFSF18, and CD28, were significantly different and highly expressed in the high-risk group.

### Chemotherapy drug analysis

**FIGURE 9:**
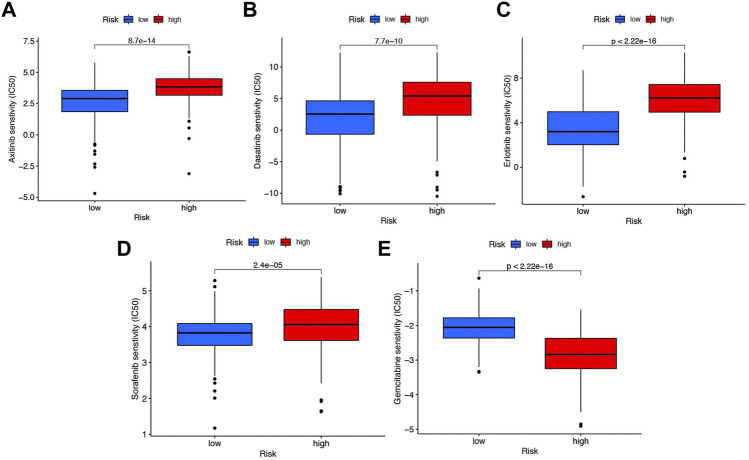
Chemotherapy drug analysis between the high- and low-risk groups in the TCGA cohort. The different drug sensitivity of **(A)** axitinib, **(B)** dasatinib, **(C)** erlotinib, **(D)** sorafenib, and **(E)** gemcitabine between the high- and low-risk groups.

With the help of the algorithm provided in the pRRophetic R package, the IC_50_ analysis of five commonly used chemotherapeutic agents for HCC was conducted in high- and low-risk groups. Here, we found axitinib, dasatinib, erlotinib, and sorafenib had great differences in estimated IC_50_ between the high- and low-risk groups; specifically, patients in the high-risk group had higher IC_50_ values, indicating that patients in the low-risk group were more sensitive to these four drugs (Figures 9A–D). Meanwhile, the IC_50_ value of gemcitabine in the low-risk group was higher than that in the high-risk group, indicating that patients belonging to the high-risk group were more sensitive to gemcitabine (Figure 9E).

### Association of consensus clustering of 6 prognostic NRlncs with characteristics, survival, and immune analysis of hepatocellular carcinoma patients

**FIGURE 10:**
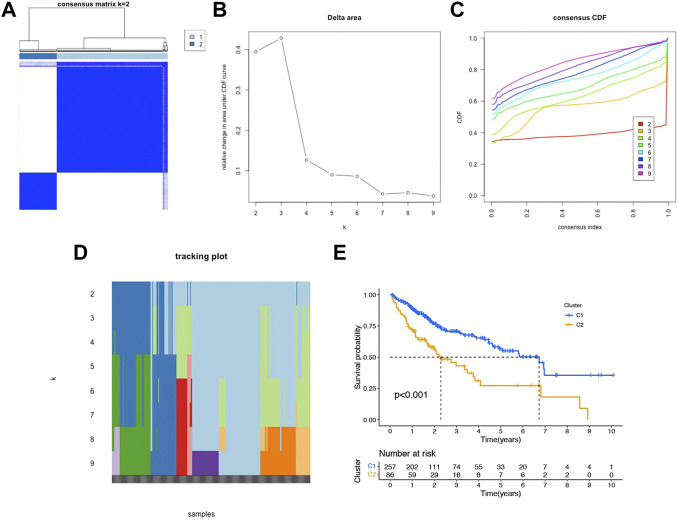
Consensus clustering and prognostic analysis based on six NRlncs. **(A)** Two different gene clusters (k = 2). **(B)** Consensus clustering cumulative distribution function (CDF) **(C)** Relative changing area under the CDF curve **(D)** Tracking plot of k from 2 to 9 **(E)** Kaplan–Meier curve of OS for two clusters in HCC.

**FIGURE 11:**
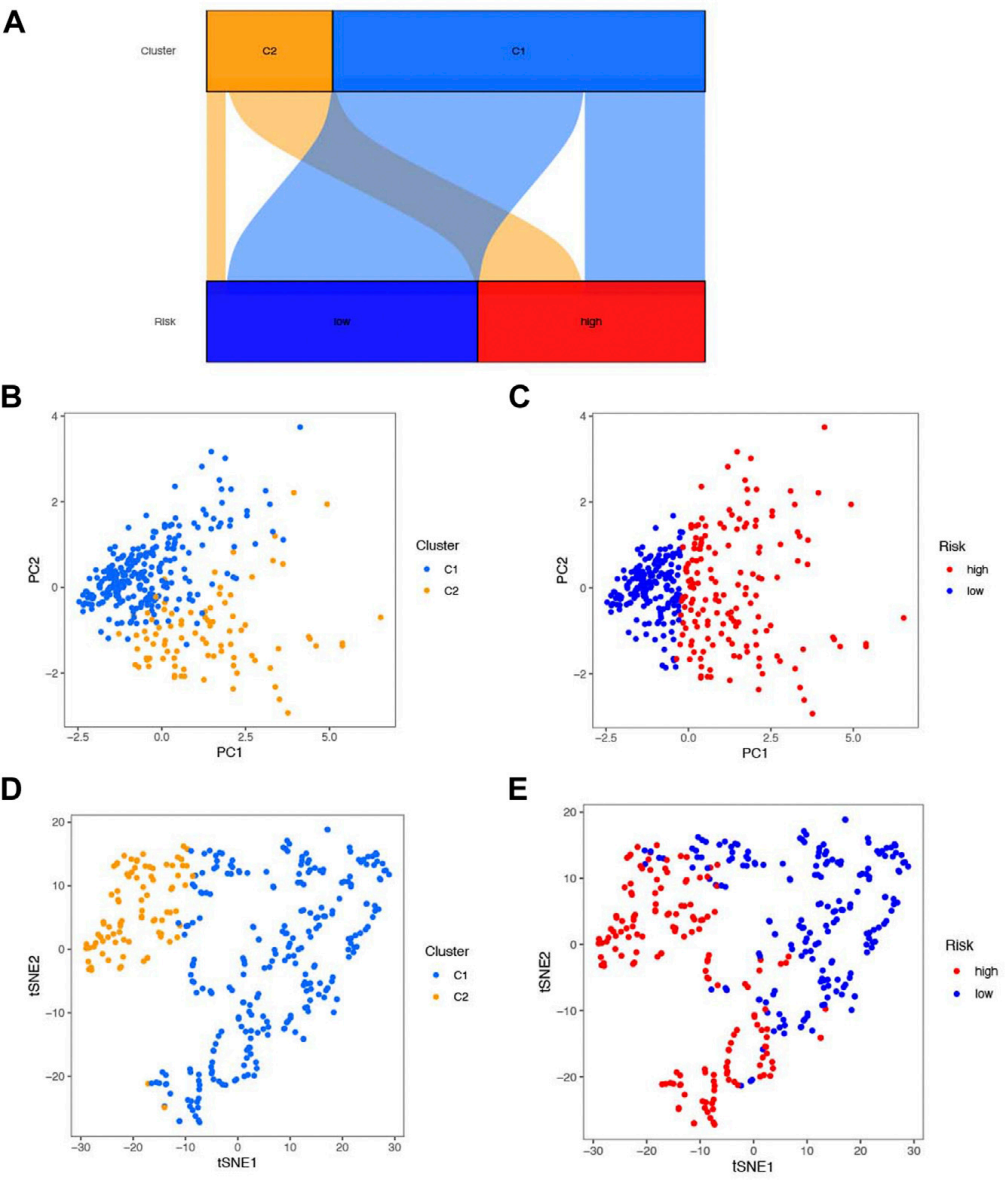
Association between classification of cluster and risk. **(A)** Sankey diagram of association between classification. **(B)** PCA score plot of clusters 1 and 2. **(C)** PCA score plot of the high- and low-risk groups. **(D)** T-SNE plot of clusters 1 and 2. **(E)** T-SNE plot of the high- and low-risk groups.

Unsupervised consensus clustering was performed to identify the potential distinct HCC clusters based on six prognostic NRlncs. As determined by the mean silhouette score, we investigated all k < 10 and found that the best clustering solution was k = 2 (Figures 10A–D). According to the clusters, the HCC patients were divided into Cluster 1 (*n* = 257) and Cluster 2 (*n* = 86). The OS in the two clusters was compared, and significant differences were reported (*p* < 0.001) (Figure 10E). In order to know how each cluster type contributed to the two groups, Sankey diagram was conducted and showed that C2 mainly belonged to the high-risk group and C1 mainly belonged to the low-risk group (Figure 11A). As shown in Figures 11B–E, PCA and *t*-SNE depicted that patients in different risk groups and clusters were mainly distributed in two directions. In addition, the trend in the relationship between classification and risk is also consistent with the Sankey diagram (Figures 11B–E).

**FIGURE 12:**
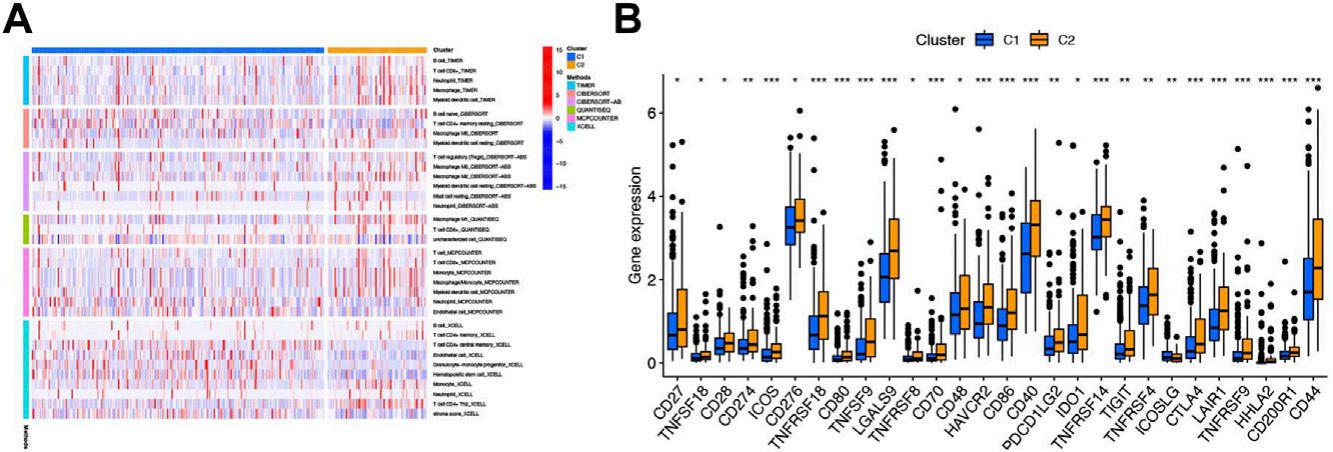
Immune-related analysis based on cluster classification. **(A)** Heatmap of immune cells in six softwares between clusters 1 and 2. **(B)** Box plot of 28 immune checkpoints between clusters 1 and 2. ***p < 0.001, **p < 0.01, and *p < 0.05.

The heatmap of immune infiltration of cluster classification based on TIMER, CIBERSORT, CIBERSORT-ABS, QUANTISEQ, MCPCOUNTER, and XCELL algorithms is shown in Figure 12A. In addition, we investigated the correlations between immune cell types expressing immune checkpoints and compared them with those in the high- and low-risk groups. Most expression trends of immune checkpoints are consistent with previous findings mentioned above, except CD48, PDCD1LG2, and ICOSLG (Figures 8D, 12B).

### Association of consensus clustering with drug analysis and validation of the prognostic NRlnc *in vitro*

**FIGURE 13:**
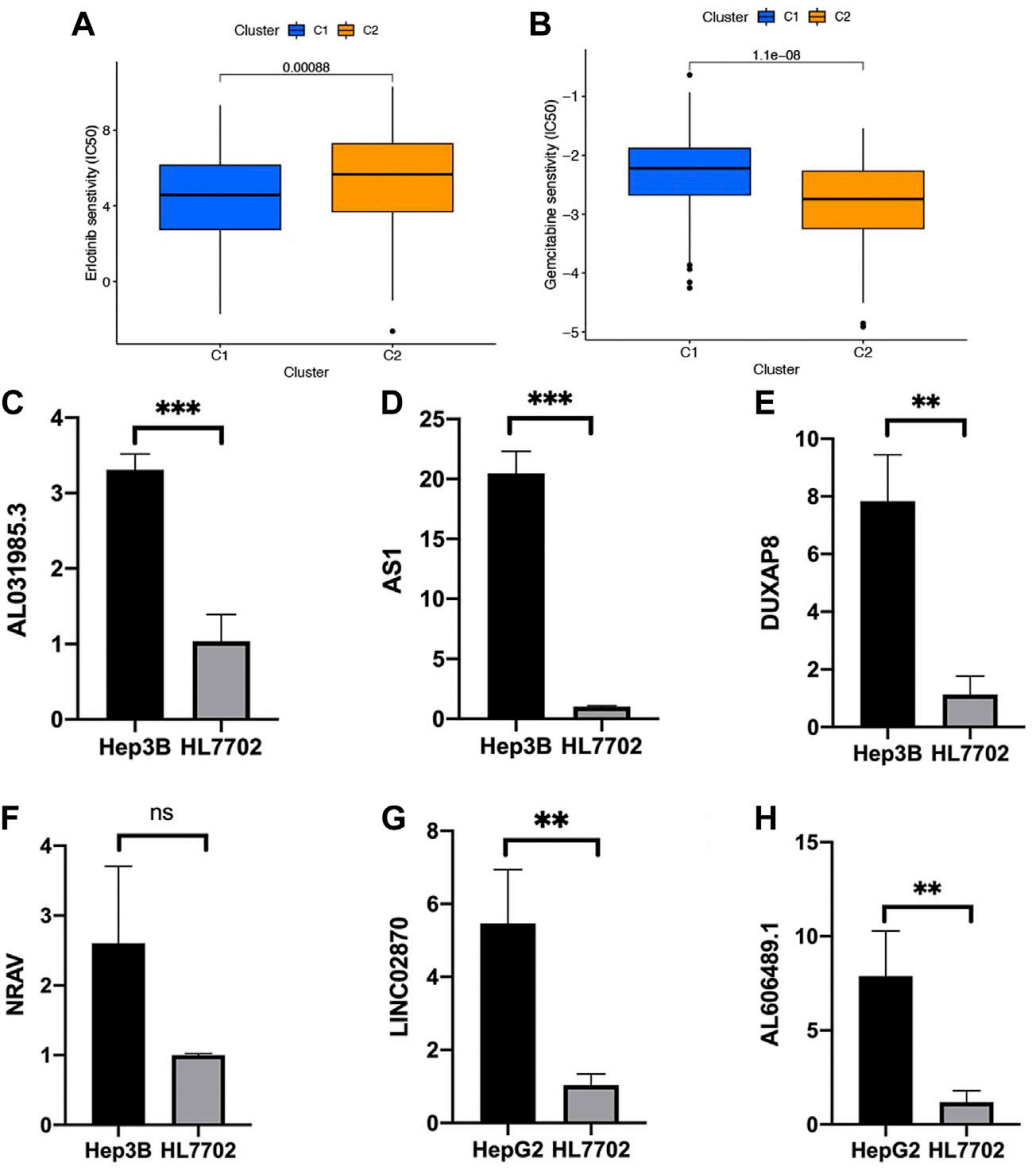
Validation of six prognostic NRlncs by qRT-PCR *in vitro*. The different drug sensitivity of **(A)** erlotinib and **(B)** gemcitabine between clusters 1 and 2. The mRNA gene expression of **(C)** AL031985.3, **(D)** ZFPM2-AS1, **(E)** DUXAP8, **(F)** NRAV, **(G)** LINC02870, and **(H)** AL606489.1 in HL7702, Hep3B, or HepG2. ***p < 0.001, **p < 0.01, ns = no significance.

We also performed the pRRophetic R package to screen for the chemotherapy drug for HCC, and several commonly drugs were screened based on the consensus clustering analysis. Among all the drugs, two kinds (erlotinib and gemcitabine) and their expression trend were consistent with the previously identified drugs based on risk classification (Figures 9A–D, Figures 13A and B). In addition, we explore the gene expression *in vitro* in Hep3B, HepG2, and HL7702. Here, we first conducted qRT-PCR on Hep3B and HL7702 and found the four NRlncs, namely, AL031985.3, ZFPM2-AS1, DUXAP8, and NRAV were all upregulated in Hep3B compared to HL7702 (Figures 13C–F). However, because of the repeated disturbance of primer polymer in Hep3B when detecting the gene expression of LINC02870 and AL606489.1, we changed another HCC cell line, named HepG2, for further research and found also to be upregulated in HepG2 compared to HL7702 (Figures 13G and H).

**FIGURE 14:**
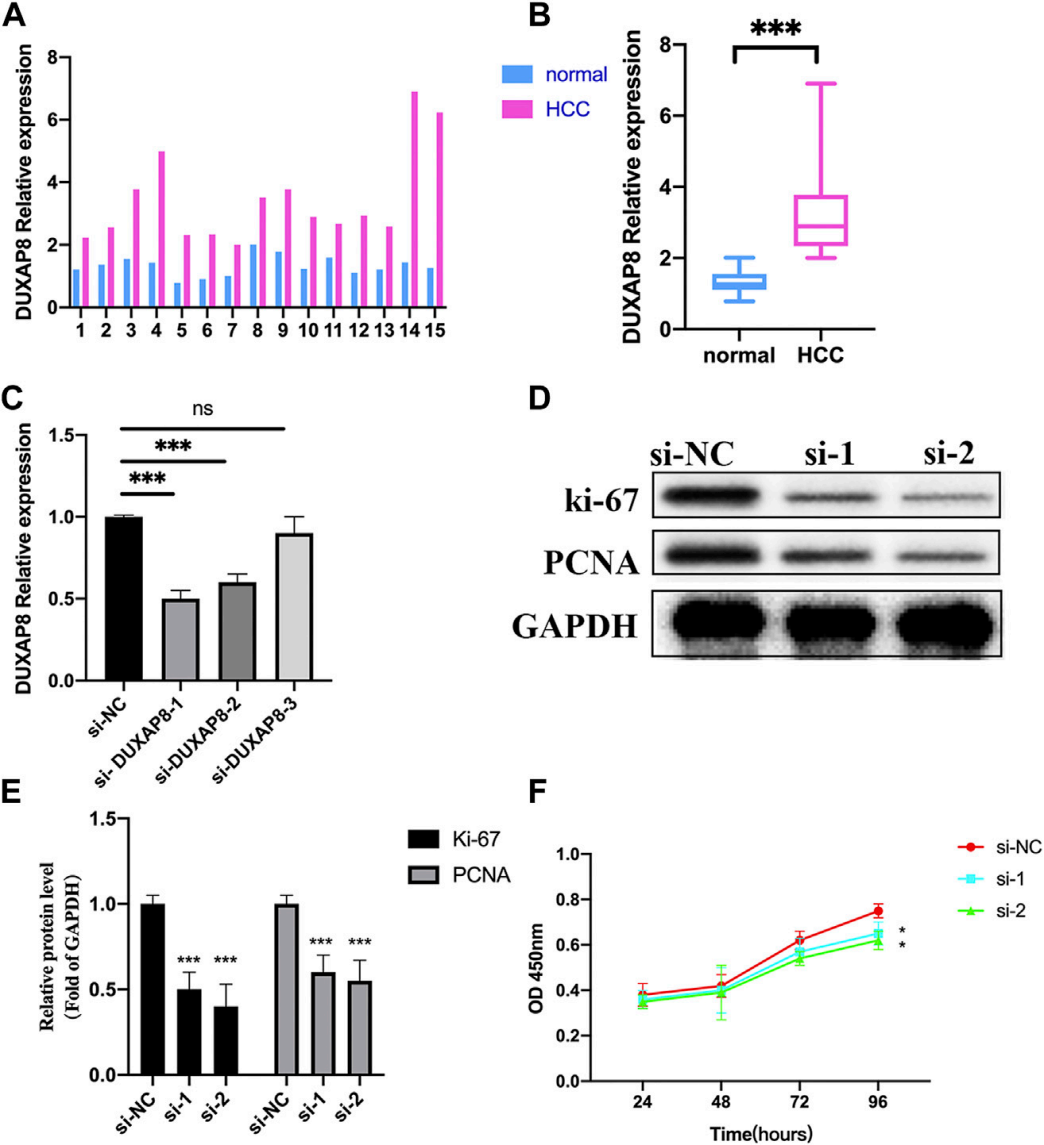
Effects of lncRNA *DUXAP8* on Hep3B cell proliferation. **(A,B)** Expression levels of DUXAP8 in 15 samples of the HCC and normal tissue by qRT-PCR. **(C)** Validation of three si-*DUXAP8* RNAs’ effects on *DUXAP8* expression levels in Hep3B cell lines. **(D)** Proliferation-related gene expression in different treated groups. **(E)** Densitometric quantification levels of proliferation-related genes in different treated groups. **(F)** Cell viability of si-1 and si-2 compared to si-NC by the CCK-8 assay. ***p < 0.001, *p < 0.05, ns = no significance.

Here, we first validated the expression of *DUXAP8* in 15 HCC samples and normal tissues, and we noticed the lncRNA expressions of *DUXAP8* were highly expressed in the HCC samples (Figures 14A and B). Then, we further validated the function of *DUXAP8* in Hep3B. After several siRNAs of *DUXAP8* were transfected, we noticed si-1 and si-2 worked well in the downregulating *DUXAP8* expression (Figure 14C). Then, the proliferation-related gene expressions of *ki-67* and *PCNA* were also downregulated after the *DUXAP8* expression was inhibited (Figures 14D and E). Also, si-1 and si-2 could also significantly inhibit the cell viability of Hep3B in 96 h (Figure 14F).

## Discussion

Abnormal expression of lncRNA can be used as a molecular biomarker for disease diagnosis, prognosis, or therapy stratification. Tumor invasion, migration, proliferation, apoptosis, and drug resistance were reported to have a connection with lncRNA. Most significantly, lncRNA, especially necroptosis-related lncRNA, participated in the occurrence and progression of HCC, which still lacks efficient treatment despite the development of surgical resection, liver transplantation, chemotherapy, immunotherapy, and targeted therapy for HCC. Necroptosis, also named programmed necrosis, was identified as a new form of cell death which is characterized by increasing inflammation of cells. Necroptosis was also reported to play an essential role in progression of various cancers, while not too many studies focused on regulation of NRlnc expression in HCC. In this research, we aimed to explore the relationship between NRlnc and their association with the outcome of HCC patients by establishing a novel prognostic signature of six NRlncs, investigating its underlying molecular mechanisms, evaluating its clinical significance in predicting disease progression, and finding the best curative treatment option.

In our study, we investigated 60 pyroptosis gene association with lncRNA and constructed a novel 6-NRlnc prognostic model of HCC. The six-NRlnc expressions and the median risk scores could relatively divide the entire patients into the high- and low-risk groups in the TCGA dataset, training cohort, and testing cohort, which indicated the six NRlncs have the ability to classify different risk groups. Then, the survival analysis showed that the six-NRlnc signature could strongly predict the OS of patients in the training, testing, and entire cohorts. The AUC of the different clinical features demonstrated that the risk score based on signature could have the highest predictable value. Time-dependent ROC analysis found it was 0.793, 0.701, and 0.673 at 1, 3, and 5 years, respectively, in TCGA, which all indicated the model had a good predictive ability for survival of HCC patients. To the best of our current knowledge, lncRNA might be involved in lncRNA regulated-miRNA, the sensitivity of chemotherapeutic drug and immunotherapy, which all contributed to tumor progression. AL606489.1 has been involved in construction of the prognostic model in lung adenocarcinoma related to pyroptosis and autophagy (Liu and Yang, 2021; Song et al., 2021). AL031985.3, LINC02870, and NRAV were reported to have participated in the prognostic signature of HCC (Chen et al., 2021; Xu et al., 2021; Zhang et al., 2021; Zhou et al., 2021). DUXAP8 could be sponged by various microRNAs, such as miR-20b-5p, miR-584-5p, or miR-448, to play a role in proliferation of papillary thyroid carcinoma cells, HCC, and pancreatic carcinoma (Li et al., 2021; Liu et al., 2021; Pang and Yang, 2021). DUXAP8 was also found to be related to resistant to PARP inhibitors (PARPis), including olaparib, rucaparib, and niraparib, in HCC therapy (Hu et al., 2020). ZFPM2-AS1 had a positive correlation with the PD-L1 expression in non-small cell lung cancers through the JAK-STAT and AKT pathways (Wang et al., 2020). According to the research aforementioned, we could say that the six NRlncs are closely related to cancer progression and therapy. In addition, a nomogram based on the six NRlncs for predicting HCC was constructed and validated. The calibration plots demonstrated that the nomogram predicted 1-, 3-, and 5- year survival rates were consistent with those of the actual survival, which indicated that the nomogram could be provided for clinicians as a prognosis prediction tool.

We further revealed the potential biological processes and pathways of the six NRlncs through GSEA enrichment analyses. It is noteworthy that CELL_CYCLE, UBIQUITIN_MEDIATED_PROTEOLYSIS, PYRIMIDINE_METABOLISM, BASE_EXCISION_REPAIR, SPLICEOSOME, ENDOCYTOSIS, and RNA_DEGRADATION were related to the scores of patients in the high-risk group. Cell cycle is closely associated with cell reproductive capacity, and lncRNAs participate in cell cycle to regulate necroptosis in tumor (Jiang et al., 2021). Ubiquitin-dependent proteolysis toward accelerated or inhibited degradation of protein regulators of tumor suppression might confer proliferation or apoptosis in tumor cells (Fuchs, 2005; Li et al., 2011). E3 ubiquitin ligase can function as an inhibitor of necroptosis (Seo et al., 2016). Inhibition of pyrimidine synthesis might regulate the urea cycle and had been represented as a novel therapeutic strategy for HCC (Ridder et al., 2021). Some kinds of base excision repair genes, like hOGG1 and XRCC1, could increase HCC patients’ risk (Yuan et al., 2012). Inhibition of APE1, a critical element in the base excision repair pathway, induces necroptosis in non-small cell lung cancer (Long et al., 2021). Spliceosome is the ribonucleoprotein machine removing non-coding introns from precursor messenger RNAs, which could also be an actionable cancer hallmark in HCC (Lopez-Canovas et al., 2021). The inhibition of U1 snRNA expression could result in necroptosis-related cell death in Alzheimer’s disease (Cheng et al., 2017). Endocytosis, an important way for cells to obtain macromolecules and granular substances from extracellular mode, is involved in necroptosis (Pradhan et al., 2021). Some research studies noticed that improving capacity of endocytosis could significantly enhance drug delivery in HCC (Saha et al., 2017). RNA degradation is essential for gene regulation, and lncRNAs can affect small RNA activity by triggering small RNA degradation (Ulitsky, 2018). Various metabolic processes (FATTY_ACID_METABOLISM, RETINOL_METABOLISM, DRUG_METABOLISM_CYTOCHROME_P450, GLYCINE_SERINE_AND_THREONINE_METABOLISM, and TRYPTOPHAN_METABOLISM), amino acid degradation (VALINE_LEUCINE_AND_ISOLEUCINE_DEGRADATION), and PRIMARY_BILE_ACID_BIOSYNTHESIS were more prominent in the low-risk group. Metabolic processing has an important role in tumor progression, which can promote the conversion of carbon into biological macromolecules by activating metabolic pathways (Boroughs and DeBerardinis, 2015). It is worth noting that alterations of cytochrome P450 in cancer may influence the metabolism of chemotherapeutic drugs, in which the related enzymes may influence treatment effect in cancer patients (Stipp and Acco, 2021). Thus, we conduct chemotherapy drug analysis in the following research. Amino acid degradation also directly affected the activity of the tumor cells and the physiological state of HCC patients (Sivanand and Vander Heiden, 2020). Considering that the aforementioned functional pathways were more or less related to necroptosis and lncRNA, further research for the relationship between NRlncs and HCC is of great significance for understanding the disease mechanisms and potential drug development. In summary, the results suggested that the six NRlncs may promote the occurrence and development of HCC through pathways aforementioned.

To explore the correlation of immune microenvironment with patients, we used multiple algorithms to plot the heatmap of immune cell infiltration of HCC patients. Here, we found that B-cell, T-cell, macrophage, and mast cell were highly infiltrated in HCC, which were associated with tumorigenesis, progression, and metastasis (Qian and Pollard, 2010; Crespo et al., 2013; Franchina et al., 2018; Aponte-López and Muñoz-Cruz, 2020). To better understand the immune microenvironment between the two risk groups, we conducted ssGSEA analysis and found aDCs, macrophages, Th2 cell, and Treg have a stronger correlation with the high-risk group. From other research studies, the aDCs might help cirrhosis collapse into HCC (Chen et al., 2000). The aggregation of macrophages and Treg and imbalance of Th1/Th2 cell obviously promoted tumor progression and drug resistance, which was consistent with our findings (Zhou et al., 2016; Zhu et al., 2019). In addition, we found the immune-related function of cytolytic activity and MHC class I were more active in the high-risk group, while Type I and Type II IFN responses were more active in the low-risk group. Cytolytic activity was found in approximately 25% of HCCs with markers of an inflammatory response (Sia et al., 2017). The MHC class I, who was highly expressed in HCC, might be suitable and practicable to be the target cytotoxic T lymphocyte vaccine against HCC (Chen et al., 2005). Host Type I and Type II IFNs are also required for optimal therapeutic efficacy in tumor therapy (Fuertes et al., 2013; Varudkar et al., 2021). These results suggest that the six-NRlnc signature may partially reflect tumor immune cell infiltration and could provide some useful information for immunotherapy. In addition, additional evidence points toward an important role for necroptosis in immune responses *via* mediating the expression levels of immune checkpoints (Tang et al., 2020). Thus, we compared immune checkpoints’ expression between the two risk groups and discovered that differences exist remarkably, which may provide underlying therapeutic targets for treatment of HCC.

It is well-known that HCC is highly resistant to several available chemotherapy agents, administered either alone or in combination. Hence, it is very important to select appropriate chemotherapy drugs for different risk groups. In our research, patients who belonged to the high-risk group were more sensitive to gemcitabine, whereas axitinib, dasatinib, erlotinib, and sorafenib may have a better effect in patients in the low-risk group.

A consensus clustering was performed to identify potential distinct HCC clusters based on six prognostic NRlncs and revalidate the difference of bioinformatics and immune-related analyses in risk-based division. Here, we found that patients in different risk groups and clusters were mainly distributed in two directions, and C2 mainly belonged to the high-risk group, while C1 mainly belonged to the low-risk group. A drug analysis was also conducted, and erlotinib and gemcitabine were found to overlap with the previous drug analysis. In order to further determine the credibility of the lncRNA in the prognostic model, we verified the expression levels of six lncRNAs in different cell lines, including Hep3B, HepG2, and HL7702, by qRT-PCR. Here, we found all the six NRlncs were upregulated in HCC cell lines when compared to normal one, whose trends were consistent with what we found in the TCGA dataset.

## Conclusion

In conclusion, we constructed a novel prognostic model based on six NRlncs to predict the prognosis of HCC patients. The model can provide useful insights into the potential prediction of HCC prognosis and helped determine potential biomarkers that might be novel therapeutic targets for HCC.

## Data Availability

The original contributions presented in the study are included in the article/Supplementary Material; further inquiries can be directed to the corresponding authors.

## Supplementary material

SUPPLEMENTARY MATERIAL S1
67 necroptosis-related genes.

SUPPLEMENTARY MATERIAL S2
769 NRlncs were significantly differentially expressed between normal tissue and HCC.

SUPPLEMENTARY MATERIAL S3
SiRNA primer sequences.

SUPPLEMENTARY MATERIAL S4
The code related to this research.

## Abbreviations

aDCs, activated dendritic cells; AUC, areas under the curve; DAMPs, damage-associated molecular patterns; DMEM, Dulbecco’s modified Eagle’s medium; FDR, false discovery rate; GSEA, Gene set enrichment analyses; HCC, hepatocellular Carcinoma; IC50, half-maximal inhibitory concentration; K-M, Kaplan–Meier; LASSO, least absolute shrinkage and selection operator; lncRNA, long non-coding RNA; MLKL, mixed lineage kinase domain-like; NRGs, necroptosis-related genes; NRlnc, necroptosis-related lncRNA; OS, overall survival; PARPi, PARP inhibitor; RIP1, receptor-interacting serine–threonine protein kinase 1; ROC, receiver operating characteristic; ssGSEA, single-sample gene set enrichment analysis; TCGA, The Cancer Genome Atlas; Th2 cell, T helper 2 cell; Treg, regulatory T cell.
